# High-fat diet enhances cell proliferation and compromises intestinal permeability in a translational canine intestinal organoid model

**DOI:** 10.1186/s12860-024-00512-w

**Published:** 2024-04-30

**Authors:** Itsuma Nagao, Yoko M. Ambrosini

**Affiliations:** 1 Department of Veterinary Clinical Sciences, College of Veterinary Medicine, Washington State University, Pullman, WA USA; 2 Department of Veterinary Internal Medicine, Graduate School of Agricultural and Life Sciences, The University of Tokyo, Tokyo, Japan

**Keywords:** Canine, Intestinal organoids, High-fat diet, Palmitic acid

## Abstract

**Background:**

Emerging evidence underscores the responsiveness of the mammalian intestine to dietary cues, notably through the involvement of LGR5 + intestinal stem cells in orchestrating responses to diet-driven signals. However, the effects of high-fat diet (HFD) on these cellular dynamics and their impact on gut integrity remain insufficiently understood. Our study aims to assess the multifaceted interactions between palmitic acid (PA), cell proliferation, and the intestinal epithelial barrier using a canine colonoid model. Canine models, due to their relevance in simulating human intestinal diseases, offer a unique platform to explore the molecular mechanisms underlying HFD derived intestinal dysfunction.

**Results:**

Canine colonoids were subjected to PA exposure, a surrogate for the effects of HFD. This intervention revealed a remarkable augmentation of cell proliferative activity. Furthermore, we observed a parallel reduction in transepithelial electrical resistance (TEER), indicating altered epithelium barrier integrity. While E-cadherin exhibited consistency, ZO-1 displayed a noteworthy reduction in fluorescence intensity within the PA-exposed group.

**Conclusions:**

By employing canine intestinal organoid systems, we provide compelling insights into the impact of PA on intestinal physiology. These findings underscore the importance of considering both cell proliferative activity and epithelial integrity in comprehending the repercussions of HFDs on intestinal health. Our study contributes to a deeper understanding of the consequences of HFD on intestinal homeostasis, utilizing valuable translational in vitro models derived from dogs.

**Supplementary Information:**

The online version contains supplementary material available at 10.1186/s12860-024-00512-w.

## Background

The mammalian intestine exhibits responsiveness to dietary cues [1–6]. Notably, LGR5 + intestinal stem cells play a crucial role in reshaping intestinal composition in response to diet-driven signals [1, 7, 8]. These cells regulate the production of daughter stem cells and progenitor cells, which subsequently differentiate into various cell types within the intestine [9, 10]. The rise in obesity has been closely linked to elevated levels of plasmatic free fatty acids (FFAs), which in turn lead to insulin resistance [11, 12] and tissue inflammation [13, 14]. However, little is known about how the adaptation of stem and progenitor cells to pro-obesity high-fat diet (HFD) alters the potential of these cells and leads to intestinal tumor formation. Recent studies have highlighted the adverse impact of fatty acid from HFD on gut homeostasis and intestinal epithelial function [1, 7]. Of particular interest is palmitic acid (PA), a prevalent saturated fatty acid in HFD made from animal fat [15, 16], renowned for its lipotoxic effects across various organs [17–20]. Notably, PA has been associated with compromised gut insulin sensitivity [21] and gut barrier function [22]. Additionally, prolonged exposure to PA has been shown to hinder cell differentiation in different tissues [7, 23, 24].

Despite growing recognition of the relationship between metabolic disorders and intestinal cell dysfunction, the underlying molecular mechanisms remain poorly elucidated [25, 26]. This knowledge gap can be attributed to the dearth of translational animal and in vitro models specifically tailored to study obesity's impact on intestinal physiology. In this context, canine models have gained prominence due to their relevance as a spontaneous, large animal model for various human intestinal diseases [27–33].

In canines subjected to a HFD, biopsies revealed a notable augmentation in stem cell characteristics [34]. Intriguingly, employing PA as a surrogate for mimicking the effects of a HFD yielded similar results, with elevated stemness characteristics coinciding with heightened expression of the peroxisome proliferator‐activated receptor‐γ, a pivotal nuclear receptor transcription factor [34]. Additionally, consumption of a HFD was reported to decrease the gut barrier function in dogs, which is consistent with findings in humans and mice [35]. Hence, Investigation of the effects of HFD on intestinal function in dogs can be expected to serve as a model for HFD in humans to bridge the knowledge gap.

This study aims to address this gap by utilizing canine intestinal organoid systems. These systems have been developed to directly assess how nutritional factors, particularly palmitate-induced effect, impact both the development of stem cell populations and the integrity of the intestinal epithelial barrier. Through this proof-of-concept investigation, we endeavor to shed light on the intricate interplay between palmitate-induced effect, stem cell dynamics, and epithelial barrier function in the context of HFD-indcuced intestinal dysfunction.

## Results

### Palmitic acid increases organoid size and LGR5 expression in canine colonoids

In this study, PA, a well-established main component of the HFD [7, 26], was used to model the effect of HFD in vitro. First, 3D organoids were used to examine the effect of PA on the proliferative activity of intestinal organoids following PA exposure. Canine colonic organoids, also known as colonoids, were cultured in organoid medium. This culturing process was maintained from the point of passaging until day 4, allowing the organoids to reach a suitable level of maturity. Subsequently, the medium was substituted with PA-containing medium, and the organoids were incubated for a duration of 24 h.

**Fig. 1:**
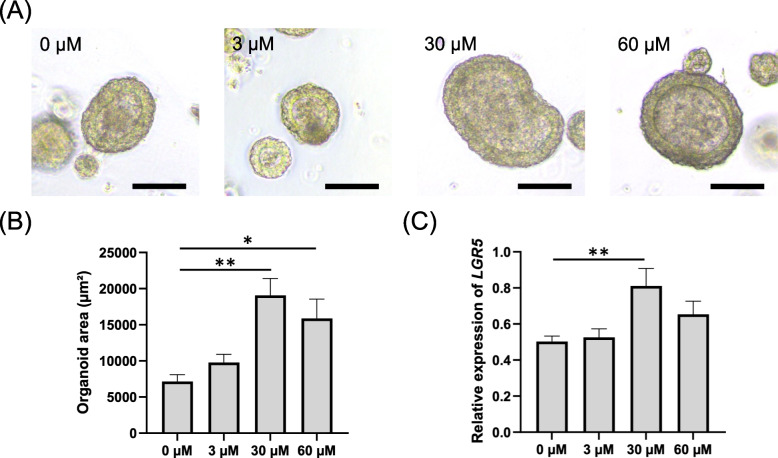
Revealing enlarged dimensions and enhanced LGR5 expression in palmitic acid-exposed 3D canine colonoids. **A** Representative phase contrast brightfield microscopy depicting 3D colonoids cultured in Matrigel following to 24 h of exposure to varying concentrations of palmitic acid (3, 30, 60 µM). For 0 µM, a carrier control consisting of 0.1% dimethyl sulfoxide (DMSO) in the media was used. Scale bar = 100 µm. **B** The organoid area (µm.^2^) was quantified from five randomly selected fields for each condition, encompassing a minimum of ten organoids, as assessed using ImageJ. This assessment was carried out across three biological replicates, each with three technical replicates. The error bars depict the standard error of the mean. ** *p* < 0.01, * *p* < 0.05. **C** The gene expression level of the stem cell marker *LGR5* in colonoids exposed to varying concentrations of palmitic acid was quantified using qPCR. Each dataset was compiled from three biological replicates, each with three technical replicates. The error bars represent the standard error of the mean. ** *p* < 0.01

Figure 1A and B demonstrate the alteration in organoid dimensions following exposure to PA. At concentrations of 30 µM and 60 µM, PA elicited a notable impact on organoid size. Specifically, the organoid dimensions increased by 2.66-fold at 30 µM (*p* < 0.01) and 2.22-fold at 60 µM (*p* = 0.02) in comparison to the control group, suggesting increased cell proliferation in colonoid exposed to PA.

In order to explore the underlying cause of the altered organoid size, we conducted an investigation into whether increased stem cell pupulation played a role. The gene expression of *LGR5*, a prominent stem cell marker, using quantitative reverse transcription polymerase chain reaction (qPCR) (Fig. 1C) was performed. Notably, the expression level of *LGR5* was found to be significantly increased by exposure to 30 µM PA (1.61 times: *p* < 0.01), although such an increase was not observed at 60 µM (*p* = 0.26). Building upon these observations, we established 30 µM as the dosage that manifests a representative effect of PA. This aligns with earlier in vivo and in vitro investigations involving mice and dogs, as evidenced by previous studies [7, 34]. Consequently, we opted to employ 30 µM PA for subsequent experiments.

### Influence of palmitic acid on the proliferative activity of canine colonoids

**Fig. 2:**
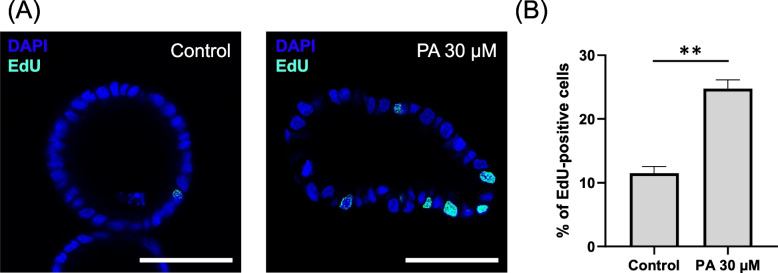
Assessment of proliferative activity in 3D canine colonoids exposed to palmitic acid. **A** Representative confocal microscopy images displaying 3D colonoids cultivated within Matrigel following a 24-h exposure to 30 µM of palmitic acid. Actively proliferating cells were marked by EdU staining (Cyan Blue), accompanied by nuclear counterstaining (DAPI; Blue). The left panel presents the image following 24 h of exposure to the carrier control (DMSO), while the right panel depicts the image after 24 h of exposure to 30 µM of palmitic acid. Scale bar = 25 µm. **B** The percentage of EdU-positive cells per nucleated cells in each condition was quantified using ImageJ. Each dataset was compiled from three biological replicates, each with three technical replicates. For each condition, five randomly chosen fields of view at 40 × magnification were selected from each sample, evaluating a minimum of ten organoids. The error bars represent the standard error of the mean. ** *p* < 0.01

To demonstrate increased cell proliferative activity of colonoids, we employed 5-ethynyl-2-deoxyuridine (EdU) assay to assess the proliferative activity of colonoids. Following a 24-h exposure to 30 µM PA, an EdU assay was conducted. The rate of EdU-positive cells exhibited a significant increase upon PA exposure (2.22 times: *p* < 0.01), providing conclusive evidence of PA-induced heightened proliferative activity (Fig. 2A and B).

### Palmitic acid exposure and its impact on intestinal barrier integrity

Subsequently, we examined the influence of PA on intestinal barrier function. To achieve this, a colonoid-derived monolayer was established by seeding single cells enzymatically isolated from Matrigel-grown colonoids onto a cell culture insert, a method previously documented [36]. These organoid-derived monolayers were cultured in organoid medium until day 5, a point at which the transepithelial electrical resistance (TEER) value reached a stable state (Supplemental Fig. 1) [36]. On day 5, the apical medium was substituted with a PA-containing medium, and the assessment of epithelial barrier integrity was conducted 24 h subsequent to this medium alteration.

**Fig. 3:**
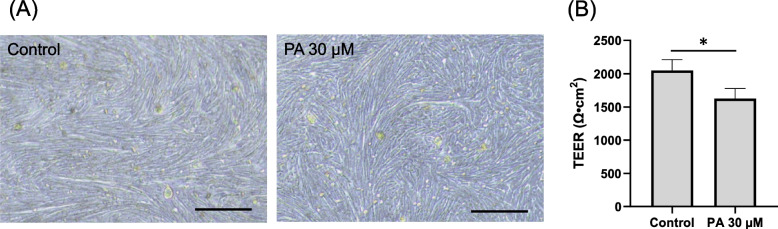
Evaluation of epithelial barrier integrity in canine colonoid-derived monolayers. **A** Representative phase contrast brightfield microscopy images of colonoid-derived monolayers cultured on cell culture inserts following a 24-h exposure to palmitic acid. The left panel presents the image following 24 h of exposure to the carrier control (DMSO), while the right panel depicts the image after 24 h of exposure to 30 µM of palmitic acid. Scale bar = 100 µm. **B** The intestinal epithelial barrier integrity was assessed through the measurement of TEER values. TEER values were recorded after treating with media containing the carrier control (DMSO) or 30 µM palmitic acid for 24 h. This assessment encompassed three biological replicates, each with three technical replicates. The error bars represent the standard error of the mean. * *p* < 0.05

While no overt disparity in cellular morphology was discernible between the Control and PA groups, as observed through phase contrast microscopy (Fig. 3A), a significant reduction in TEER value was evident after a 24-h PA exposure (0.79 times: *p* = 0.02) (Fig. 3B), indicating gut barrier integrity was decreased by addition of PA.

### Palmitic acid exposure leads to downregulation of tight junction protein expression

To elucidate the underlying cause behind the observed decline in epithelial barrier integrity, substantiated by TEER measurements, the expression levels of E-cadherin and Zonula occludens-1 (ZO-1) were evaluated by immunocytochemistry. These are proteins that play a crucial role in maintaining the integrity and function of cell–cell junctions, particularly in epithelial tissues [37, 38].

**Fig. 4:**
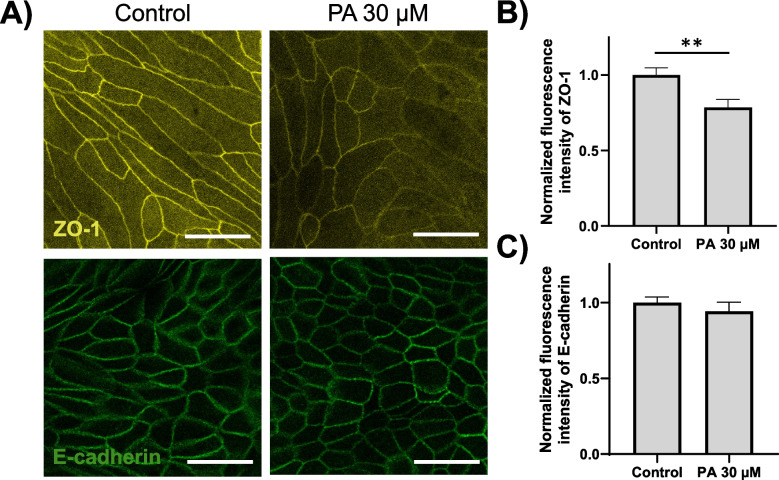
Tight junction proteins in canine colonoid-derived monolayers. **A** Representative immunocytochemistry image depicting tight junction proteins (ZO-1: Yellow, E-cadherin: Green) within a colonoid-derived monolayer treated with media containing the carrier control (DMSO) or 30 µM palmitic acid. The left panel represents the control group, and the right panel depicts the palmitic acid-treated group. Scale bar = 25 µm. **B** Fluorescence intensity in the entire field of view was quantified using ImageJ and subsequently normalized by dividing each intensity by the average of the intensity of the control group. The upper panel displays the normalized fluorescence intensity of ZO-1, while the lower panel showcases that of E-cadherin. This assessment was conducted across three biological replicates, each with three technical replicates. For each condition, five randomly selected fields of view at 63 × magnification were evaluated in each sample. The error bars represent the standard error of the mean. ** *p* < 0.01

In the control group, both ZO-1 and E-cadherin displayed distinct staining patterns at cell boundaries. However, within the PA-exposed group, E-cadherin exhibited staining comparable to the control, whereas ZO-1 demonstrated a conspicuous reduction in fluorescence intensity (Fig. 4A).

The quantification of these fluorescence intensities using Image J, revealed no significant variance in fluorescence intensity concerning E-cadherin. However, a marked reduction in fluorescence intensity was detected for ZO-1 within the PA group (0.78 times: *p* < 0.01) (Fig. 4B), implying that decreased expression of ZO-1 causes a decrease in gut barrier integrity as indicated by TEER. To further examine changes in tight junction protein expression, we evaluated gene expression levels of Occludin and Junctional adhesion molecule A (JAM-A), which have been reported to be expressed in the canine intestine other than ZO-1 and E-cadherin [39, 40]. However, no significant differences between the two groups were detected in both *Occludin* and *JAM-A* (Supplemental Fig. 2).

## Discussion

The presented results shed light on the impact of PA exposure on canine colonoids as well as on canine colonoid-derived monolayers, extending our understanding of how dietary components can influence intestinal homeostasis.

The enhanced cell proliferative activity observed in response to PA exposure prompts discussions on the potential molecular pathways and signalling mechanisms that mediate these effects. In studies using mice HFD model, there is evidence suggesting that PA exposure can influence stemness in the intestinal epithelium [7]. This suggests a necessity for additional experimentation on canine organoids, particularly through colony formation assays, to more accurately evaluate and quantify any enhancement in stem cell function and the population of stem cells with self-renewal abilities following PA treatment. Elevated levels of PA have been correlated with alterations in the intestinal stem cell niche and increased proliferation of stem cells [7]. This may have implications for tissue repair and regeneration but could also contribute to hyperproliferative disorders if dysregulated. In parallel, canine investigations have revealed a similar trend where PA exposure is linked to an amplification of stemness in intestinal cells [34]. Notably, studies employing canine enteroids have disclosed that PA exposure elicits an elevation in the expression of stem cell markers such as LGR5, concurrent with heightened expression of peroxisome proliferator-activated receptor-γ, a pivotal nuclear receptor transcription factor [34]. Studies on mice have demonstrated an upregulation of peroxisome proliferator-activated receptor-δ (PPAR-δ) in the intestinal stem cells of those on a high-fat diet (HFD) [1]. Furthermore, when mouse intestinal organoids are treated with PPAR-δ agonists, an enhancement in stemness is observed, mirroring that seen in organoids derived from HFD-fed mice [1]. By integrating these findings with observations from dogs on a HFD, we propose that a similar upregulation of peroxisome proliferator-activated receptors might contribute to the increased proliferative activity observed in canine colonoids. This suggests a potential molecular pathway through which PA exposure may influence stemness and intestinal barrier integrity, highlighting the relevance of PPAR-δ pathways in the observed phenotypic changes.

The compromised integrity of the intestinal barrier, resulting from exposure to PA in canine colonoid-derived monolayers, underscores the critical role of tight junction proteins in upholding epithelial integrity. Among dietary components, PA has garnered significant attention within the scientific community, primarily due to its well-established lipotoxic effects on various human organs. Notably, our study aligns with previous research, revealing the growing understanding of PA's impact on intestinal barrier function [41]. This underlines the complexity of its effects and lends credence to our current findings showcasing compromised intestinal barrier integrity following PA exposure. The consistent outcomes further accentuate the potential of utilizing dogs as a valuable in vitro model to explore human intestinal homeostasis. However, the cause of the reduction of gut barrier integrity by HFD remains to be elucidated. Some reports indicate that HFD-induced oxidative stress causes a decrease in tight junction proteins in the intestinal tract [15, 42], while other reports indicate that tight junction proteins are decreased in HFD models even though oxidative stress is not induced [41]. Exploring the cause which tight junction protein levels decrease in a human-relevant canine colonoid model could uncover the factors behind the decline in gut barrier integrity linked to HFD in humans.

The congruence between our observations in canine colonoids and the findings from in vivo human [15, 43] and dog studies [34, 35] bolsters the translational significance of our research. The consistent outcomes observed across diverse models emphasize the potential relevance of our findings to human health, aligning with the principles of the One Health initiative. In humans, feeding a HFD is known to impair intestinal function or to be involved in tumor formation, but the molecular mechanisms of the effects of a HFD on the intestinal epithelium have not yet been fully elucidated due to the lack of appropriate in vitro models. Although HFD has been studied in vitro in mice using intestinal organoids [7, 26], mice and humans differ greatly in their living environment, diet, and intestinal microflora composition, and there is a large gap as an animal model [44]. Therefore, we examined the direct interaction between PA and intestinal epithelial cells using canine intestinal organoids, whose living environment is close to that of humans and whose intestinal microbiota composition is relatively similar to that of humans [44, 45]. Hence, the canine in vitro HFD model in this study is considered to be an important tool for elucidating the mechanism of intestinal dysfunction related to HFD not only in dogs but also in humans.

While our canine colonoids and colonoid-derived monolayers offer a valuable platform for nutritional assays aimed at investigating early events in the intestinal alterations induced by HFD consumption, it is important to acknowledge the limitations inherent in our models. Notably, our in vitro models comprise various cell types, yet they only consist of intestinal epithelial cells. The intricate maintenance of intestinal homeostasis involves a complex interplay among multiple key players, including the intestinal microbiome, local immune cells, and intestinal epithelial cells [46].

It is imperative to recognize that PA, though an important dietary component, represents just one facet of the diverse array of fatty acids present in HFD. Our study's focus on PA offers insight into specific mechanisms, but future research should explore the effects of other fatty acids commonly found in HFD [15]. This expanded investigation could provide a more comprehensive understanding of the multifaceted impact of dietary fat on intestinal function.

Moreover, the exploration of more advanced in vitro models, such as Gut-on-a-Chip systems [47, 48], presents a new avenue for research. These systems can better mimic the complexity of the intestinal environment by incorporating aspects like fluid flow, mechanical forces, and cellular interactions [48]. Integrating these elements could enhance our understanding of how dietary factors interact with the gut milieu.

## Conclusion

In conclusion, this study demonstrated that exposure to PA has diverse effects on canine colonoids as well as colonoid-derived monolayers, mirroring similar findings observed in mice colonoids and in vivo studies with dogs [7, 34, 35]. It increases proliferative activity and impairs intestinal barrier integrity by downregulating tight junction protein expression. These consistent findings across different experimental settings contribute to our understanding of how PA, a prevalent component of HFD, can potentially disrupt gut homeostasis and function in direct way to the gut epithelium, without the involvement of gut microbiota.

## Materials and methods

### Generation and culture of canine colonoids

Intestinal biopsies were obtained by colonoscopy from three clinically healthy dogs presented to Washington State University Veterinary Teaching Hospital Community Service for a dental procedure. These dogs were initially screened based on physical exam and blood work, no history of chronic disease (heart, kidney, liver, and intestinal) and be between 1 and 12 years old. If the dog is clear of these aspects and deem appropriate to seek the elective anesthetic procedure at Washington State University Veterinary Teaching Hospital Community Service, the dogs were considered clinically healthy. All animal procedure were approved by Washington State University Institutional Animal Care and Use Committee (IACUC approval: ASAF#6993). The signalment of these healthy dogs is listed in Supplemental Table 1. Colonic stem cells were collected and cultured as previously reported [49]. Briefly, colonic biopsies were washed using ice-cold Dulbecco’s phosphate-buffered saline (PBS, Gibco) containing 1 × Penicillin/Streptomycin (Gibco) for five times, cut into small pieces, and incubated in 30 mM EDTA solution (Invitrogen) for 60 min at 4 °C to release crypts containing intestinal stem cells. Collected intestinal crypts were suspended in Matrigel (Corning) and seeded to a 48-well plate (Thermo Scientific) in 30 µl per well. After the Matrigel dome was solidified in 37 °C incubator, 500 µl of organoid culture medium was added to each well. For composition of organoid culture medium, we followed the previous study; DMEM/F12 (Gibco) supplemented with 2 mM GultaMAX (Gibco), 10 mM HEPES (Gibco), 1 × Penicillin/Streptomycin (Gibco), 10% (vol/vol) conditioned medium of Noggin [50], 20% (vol/vol) conditioned medium of R-spondin, 100 ng/ml recombinant murine Wnt-3a (PeproTech), 50 ng/ml murine Epidermal Growth Factor (EGF) (PeproTech), 10 nM Gastrin (Sigma-Aldrich), 500 nM A-83–01 (Sigma-Aldrich), 10 µM SB202190 (Sigma-Aldrich), 1 mM N-Acetyl-L-Cysteine (MP Biomedicals), 10 mM Nicotinamide (Sigma-Aldrich), 1 × B27 supplement (Gibco), 1 × N2 MAX media supplement (R&D Systems), 100 µg/ml Pimocin (Invitrogen). 10 µM Y-27632 (Stem Cell Technologies) and 2.5 µM CHIR 99021 (Stem Cell Technologies) were added to the organoid culture medium for the first two days after the crypts were isolated. The culture medium was changed every other day, and colonoids were passaged once a week by breaking down the colonoids using TrypLE Express (Gibco), spinning down them (200 g, 5 min, 4 °C), resuspending them in Matrigel, and seeding them in a 48-well plate.

### Establishment of canine colonoid-derived monolayer culture

The 3D colonoids were harvested from Matrigel after being cultured for 4–6 days. This was achieved by dissolving the Matrigel dome using Cell Recovery Solution (Corning), following the manufacturer's instructions. The collected organoids, along with the solution, were then treated by incubating them in TrypLE Express containing 10 µM of Y-27632 for 10 min at 37 °C. Subsequently, the disrupted organoids were filtered through a 70 µm cell strainer (Fisher Scientific) and centrifuged at 200 g, 4 °C for 5 min.

For the preparation of Falcon cell culture inserts (0.4 µm pores, Falcon), a coating process was carried out using 100 µg/ml Matrigel and 30 mg/ml collagen I (Gibco) in DMEM/F12 supplemented with 2 mM GlutaMAX, 10 mM HEPES, and 1 × Penicillin/Streptomycin. This coating process took place at 37 °C for 1 h. Dissociated single cells obtained from the colonoids were then quantified using a Hemocytometer and seeded at a concentration of 10^6^ cells/ml within the pre-coated cell culture insert. The culture medium was changed every other day.

### Palmitic acid exposure in canine colonoids

In this study, we employed PA (Cayman chemical), a well-established key component of the HFD [7, 26], to simulate the effects of a HFD in an in vitro setting. To evaluate the cell proliferation of colonoids, we utilized 3D colonoids that were cultured in Matrigel domes for 4 days. These colonoids were then subjected to be exposed to organoid culture medium containing varying concentrations of PA (3, 30, 60 µM) for 24 h, duration already reported as an in vitro model of short-term HFD exposure [26, 41]. The concentrations of PA were chosen based on existing literature, where 3 µM represents a minimal effective dose [9], 30 µM approximates physiological responses to high-fat diets [1], and 60 µM assesses potential toxicity thresholds in intestinal organoid viability [10]. For the assessment of colonoid gut barrier function, a colonoid-derived monolayer culture spanning 5 days was utilized. After this period, only the apical medium was replaced with organoid culture medium containing 30 µM of PA. As a control, a carrier control consisting of 0.1% dimethyl sulfoxide (DMSO) in the media was used. Each experiment was conducted with three biological replicates and three technical replicates.

### Analysis of canine colonoids growth

After a 24-h exposure to PA, images of colonoids were captured under a phase contrast microscope (DMi1, Leica) at 10 × magnification to assess their growth rate. The surface area of the colonoids was measured using ImageJ (FIJI, http://fiji.sc/Fiji) [51]. For each condition, five fields of view were randomly selected at 10 × magnification within each well, and a minimum of ten organoids were analyzed.

### Total RNA extraction and quantitative reverse transcription polymerase chain reaction analysis

Colonoids exposed to PA were harvested by dissociating the Matrigel dome using Cell Recovery Solution. Subsequently, total RNA was extracted from the colonoids utilizing the RNeasy mini kit (Qiagen) and subjected to reverse transcription using the High-Capacity cDNA Reverse Transcription Kit (Applied Biosystems). qPCR was conducted by PowerUp SYBR Green Master Mix (Applied Biosystems) and the CFX96 Touch Real-time PCR Detection System (Bio-Rad) using 20 ng of cDNA to evaluate the gene expression levels of *LGR5*, a significant stem cell marker, *Occludin* and *JAM-A*, tight junction proteins. For cDNA normalization, internal control genes were employed, namely, *Succinate dehydrogenase complex subunit A* (*SDHA*), *Hydroxymethyl-bilane synthase* (*HMBS*), and *Hypoxanthine phosphoribosyl-transferase 1* (*HPRT1*), as previously reported in colon tissue [52]. Details of the primers used in this study can be found in Supplemental Table 2.

### Assessment of proliferative activity

To evaluate the proliferative activity of colonoids exposed to PA, we conducted a EdU assay using the Click-iT EdU Cell Proliferation Kit (Thermo Fisher Scientific), as we have previously reported [53]. Briefly, colonoids exposed to PA for 24 h were preincubated with a 10 µM EdU solution for 3 h, followed by fixation with 4% PFA (Thermo Scientific) for 15 min. Subsequently, these organoids were permeabilized using 0.3% Triton-X (Thermo Scientific) for 10 min and stained with the EdU staining solution and 4’,6-diamidino-2-phenylindole dihydrochloride (DAPI) (Thermo Fisher Scientific) for 30 min. Then, stained colonoids were mounted by Prolong Gold Antifade reagent (Thermo Fisher Scientific) onto glass-bottom dish (Matsunami). EdU-positive cells were captured using a confocal microscope (SP8-X, Leica) with excitation laser sources of 405 nm, 499 nm, 553 nm, and 653 nm and high efficiency Leica HyD detector, the acquired images were further processed using LAS X software (Leica) for analysis, and the percentage of EdU-positive cells was quantified using ImageJ software. For each condition, we selected five randomly chosen fields of view at 40 × magnification from each sample, evaluating a minimum of ten organoids.

### Assessment of intestinal barrier integrity in colonoid-derived monolayer

The intestinal barrier integrity of the colonoid-derived monolayer was assessed by measuring the TEER value following exposure to PA. Electrical resistance (Ω_t_) was quantified using Ag/AgCl electrodes connected to a Volt-Ohm meter (Millicell ERS-2, Millipore), and this value was then transformed into a TEER measurement using the following equation: TEER = (Ω_t_—Ω_blank_) x A. Here, Ω_blank_ represents the resistance of the blank well in ohms, and A denotes the surface area of the culture insert in cm^2^. For each condition, measurements were made with 3 biological replicates with 3 technical replicates.

### Immunocytochemistry of colonoid-derived monolayer

After exposure to PA, the colonoid-derived monolayer was fixed using 4% PFA at room temperature for 15 min. Following permeabilization with 0.3% Triton-X for 15 min at room temperature and blocking with 2% bovine serum albumin (Cytiva) for 30 min at room temperature, primary antibodies were applied to the colonoid-derived monolayer overnight at 4 °C. For visualization of E-cadherin and ZO-1, monoclonal anti-mouse E-cadherin antibody (36/E-cadherin, dilution 1:200, BD Biosciences) and polyclonal anti-rabbit ZO-1 antibody (61–7300, dilution 1:50, Invitrogen) were utilized as primary antibodies. Subsequently, the colonoid-derived monolayers were washed with PBS and incubated with secondary antibodies (Anti-Rabbit IgG H&L labeled with Alexa Fluor 555, dilution 1:1000, Abcam). After another round of washing, nuclei and F-actin were stained using DAPI and Alexa Fluor 647 Phalloidin (Thermo Fisher Scientific), respectively. The monolayer was then mounted with Prolong Gold Antifade reagent (Thermo Fisher Scientific) and imaged using a confocal microscope (SP8-X, Leica) using 63 × magnification with excitation laser sources of 405 nm, 499 nm, 553 nm, and 653 nm and high efficiency Leica HyD detector, the acquired images were further processed using LAS X software (Leica) for analysis. For each condition, five randomly selected fields of view at 63 × magnification were evaluated in each sample, and fluorescence intensity in the entire field of view was quantified using ImageJ software. Subsequently, the normalized fluorescence intensity was calculated by dividing the fluorescent intensity of each sample by the average of fluorescence intensity of control wells.

### Statistical analysis

Statistical analysis was conducted using R Studio v1.4.1717 (RStudio) and figures were generated using GraphPad Prism 10.0.2.232 (Dotmatics). To confirm the normality of each dataset, the Shapiro–Wilk's test was performed. The Kruskal–Wallis and Dunnett tests were employed for the comparison of organoid size and *LGR5* gene expression among different conditions. The Wilcoxon test was utilized to compare the rates of EdU-positive cells and TEER values across conditions. Additionally, Student t-tests were applied to compare the fluorescence intensity of tight junction proteins. All results were presented as mean ± standard error of the mean. A significance level of *p* < 0.05 was considered statistically significant.

### Supplementary Information

**Supplementary Material 1.**

## Data Availability

All relevant data are within the paper and its Supporting Information files.

## Abbreviations

EdU: 5-Ethynyl-2-deoxyuridine
FFAs: Free fatty acids
HFD: High-fat diet
HMBS: Hydroxymethyl-bilane synthase
HPRT1: Hypoxanthine phosphoribosyl-transferase 1
PA: Palmitic acid
PBS: Phosphate-buffered saline
qPCR: Quantitative reverse transcription polymerase chain reaction
SDHA: Succinate dehydrogenase complex subunit A
TEER: Transepithelial electrical resistance
ZO-1: Zonula occludens-1
