# Supplementary material for: High-fat diet enhances cell proliferation and compromises intestinal permeability in a translational canine intestinal organoid model
Source: BMC Mol Cell Biol. 2024 Apr 30;25:14. doi: 10.1186/s12860-024-00512-w (PMC11059635; doi:10.1186/s12860-024-00512-w)
Supplement: Supplementary file 1 — Supplementary Material 1. [file 12860_2024_512_MOESM1_ESM.docx]

**Supplementary figure & table Information**

**
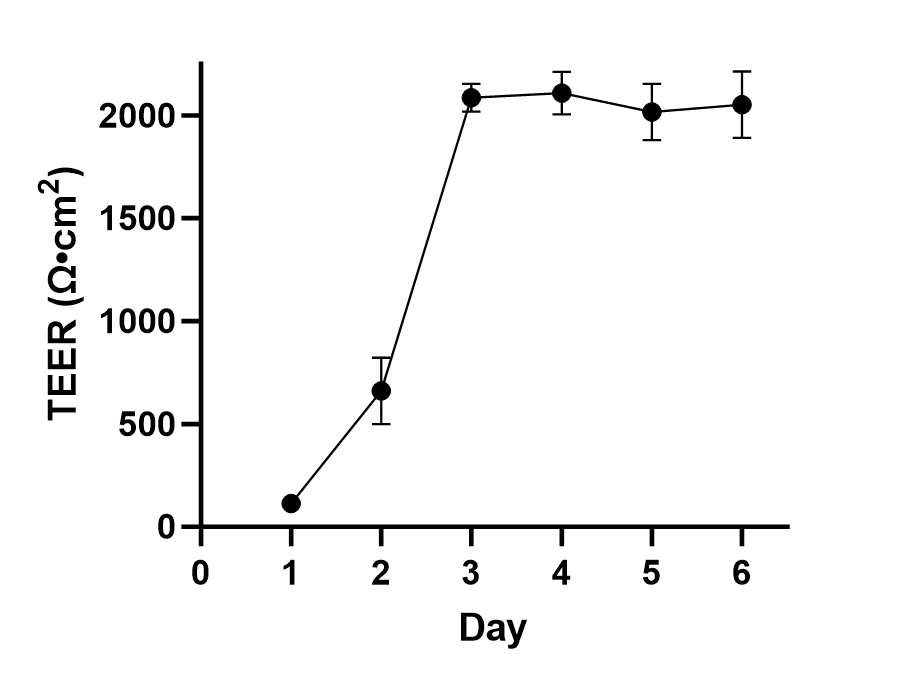
**

**Supplemental Figure 1. Change in transepithelial electrical resistance value over time of canine colonoid-derived monolayer.**

Transepithelial electrical resistance value was measured every day after seeding single cells onto cell culture insert. This assessment was conducted across three biological replicates, each with three technical replicates. The error bars represent the standard error of the mean.

**Supplemental Figure 2. Changes in gene expression levels of tight junction proteins.**

Gene expression levels of *Occludin* and *Junctional adhesion molecule A* (*JAM-A*), which are tight junction proteins expressed in canine intestine were measured by qPCR in colonoid-derived monolayer exposed to 30 µM of palmitic acid for 24 hours. Each dataset was compiled from three biological replicates, each with three technical replicates. The error bars represent the standard error of the mean.

**Supplemental Table 1. Signalment of healthy dogs included this study**.

Signalment (breed, sex, and age) of healthy dogs used in this study is summarized.

**Supplemental Table 2. Primer information.**

The information for the primers used for this study is listed.
